# Infant Oral Mutilation

**DOI:** 10.1155/2018/7586468

**Published:** 2018-02-21

**Authors:** Emily A. Pope, Michael W. Roberts, E. LaRee Johnson, Clark L. Morris

**Affiliations:** ^1^University of North Carolina School of Dentistry, Chapel Hill, NC, USA; ^2^Department of Pediatric Dentistry, University of North Carolina School of Dentistry, Chapel Hill, NC, USA; ^3^Department of Pediatric Dentistry, Private Practice of Pediatric Dentistry, Raleigh, NC, USA; ^4^Private Practice of Pediatric Dentistry, Raleigh, NC, USA

## Abstract

*Ebinyo* refers to the practice of removing primary canine tooth follicles in infants without anesthetic by African traditional healers or elders using unsterilized instruments. This report describes a case of *ebinyo* or infant oral mutilation (IOM) and associated sequelae in a child adopted from a remote African tribe. The intraoral examination revealed that the patient was missing his primary maxillary and mandibular canines. The maxillary anterior periapical radiograph displayed a dysmorphic ectopic unerupted maxillary right primary canine positioned mesial to the maxillary right primary first molar. Periapical films taken confirmed partial or complete absence of the patient's primary mandibular left (73) and mandibular right (83) canines, and a bitewing and periapical film confirmed the absence of the patient's primary maxillary left (63) canine. The permanent canines will be monitored for possible hypoplasia secondary to trauma to the tooth buds during extirpation of the primary canines. Research presented in this report reveals that there are serious health implications involved with the practice of *ebinyo*.

## 1. Introduction

Celebrities like Madonna and Angelina Jolie have adopted children from Africa and apparently many others have also. Adoptions from Africa, as a whole, have risen worldwide from 5% in 2003 to 22% in 2009, with Ethiopia ranking second behind China in international adoptions [1]. This pattern of international adoptions, paired with unique cultural practices in the child's place of birth, will present novel challenges to healthcare professions involving customs and traditions with which they may not be familiar.

In Africa, traditional healers are part of a longstanding custom and tradition. These healers can be herbalists, faith healers, or diviners that are able to communicate with ancestral spirits for diagnostic assistance, but are not usually recognized by the government [2]. Thus, they have little interaction with the healthcare system, leading to practices that are often in conflict with western medicine [3].

Many Africans seek traditional healers for a variety of reasons, ranging from immunization against witchcraft to the treatment of sexually transmitted diseases. In fact, 80% of the African population utilizes traditional healers for medical advice and treatment [4]. Data from one study, collected from 30 traditional healers and 300 of their patients, revealed that 70% of patients turn to traditional healers as their first choice for medical advice [5].

With limited access to modern medical care, especially in remote tribal villages, traditional healers are often more affordable and easier to access. In South Africa, there are approximately 200,000 traditional healers compared to only 25,000 medically trained physicians. As this disparity pertains to dental medicine, the World Health Organization reports that, in Ethiopia, 93 dentists serve more than 77 million people [6]. This disproportion suggests that many people have few options but to rely on tribal elders and healers for dental as well as medical advice.

This report describes a case of infant oral mutilation (IOM) and associated sequelae in a child adopted from a remote African tribe and provides a context in which to understand the clinical findings.

## 2. Case Report

A 4-year-old African male was referred to a private pediatric dental practice for an evaluation. The child was reported to have been adopted at nine months of age. By history, the patient's primary canines had been extracted prior to three months of age. According to the adoptive mother, the child had no outstanding health concerns. Beyond canine extirpation, all health history information prior to the time of the adoption, as well as family history, was unknown.

**Figure 1:**
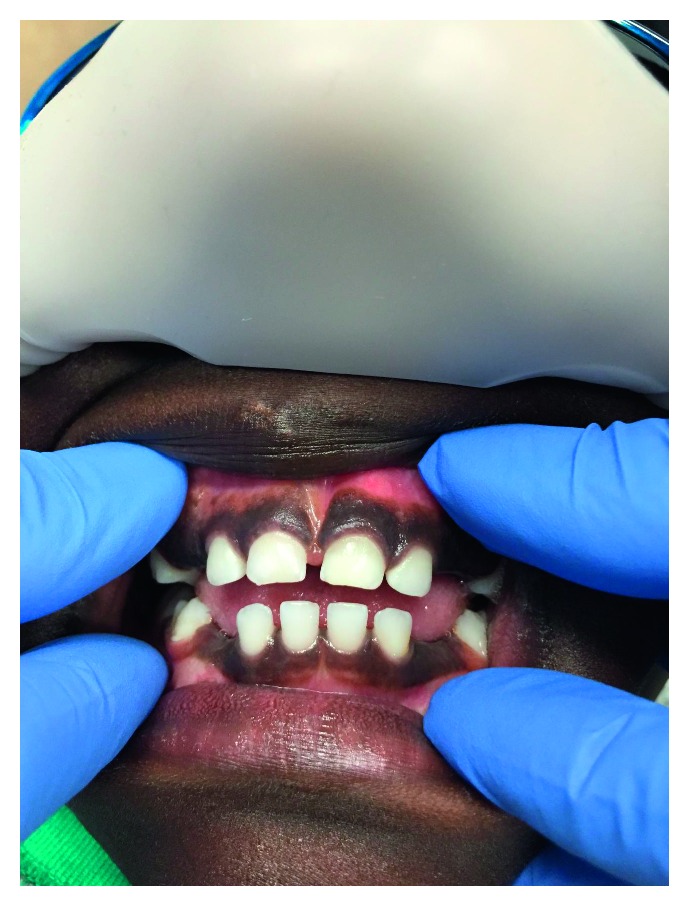
An intraoral photo of the 4-year-old male patient shows the missing maxillary and mandibular right and left primary canines.

Upon evaluation, it was noted that the patient was missing the maxillary and mandibular right and left primary canines (Figure 1). Following the clinical evaluation, two bitewings in addition to maxillary and mandibular anterior periapical radiographs were obtained to evaluate proximal tooth surfaces for caries, investigate canine areas, and rule out other pathology.

**Figure 2:**
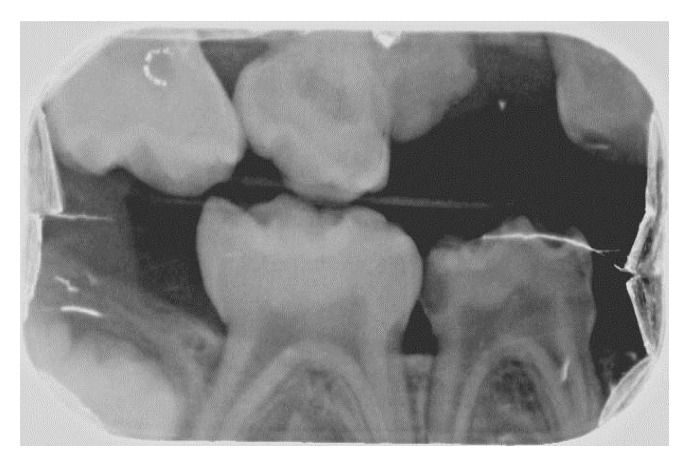
The right bitewing radiograph shows a suspicious area mesial to the maxillary right first primary molar. Caries are noted on the distal surface of the mandibular right first primary molar.

**Figure 3:**
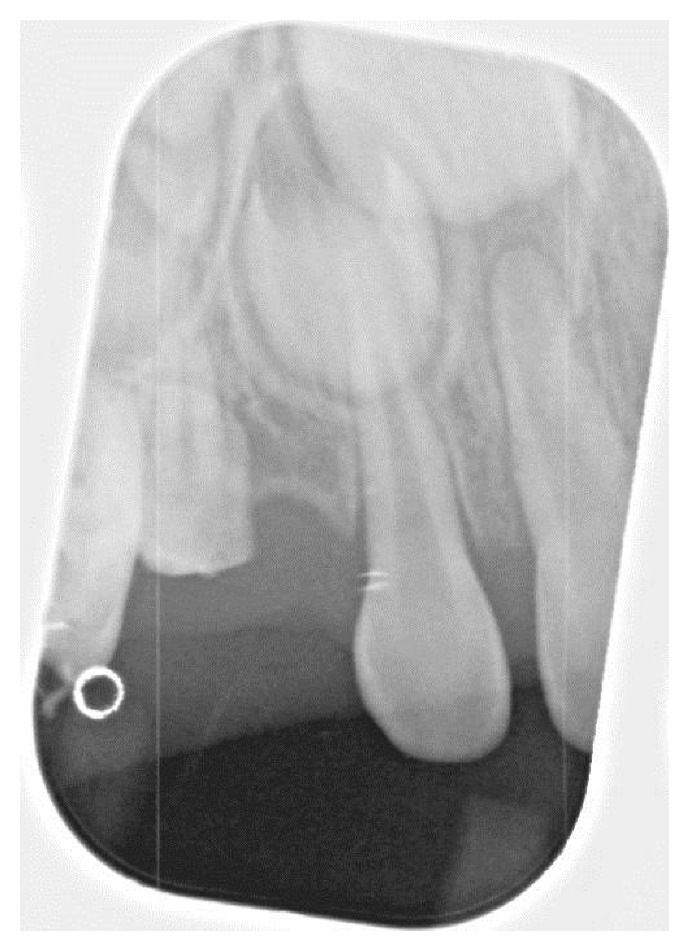
This radiograph demonstrates a dysmorphic maxillary right primary canine erupting ectopically into the mesial aspect of the maxillary right first primary molar. This is probably the result of incomplete extirpation by a tribal leader.

**Figure 4:**
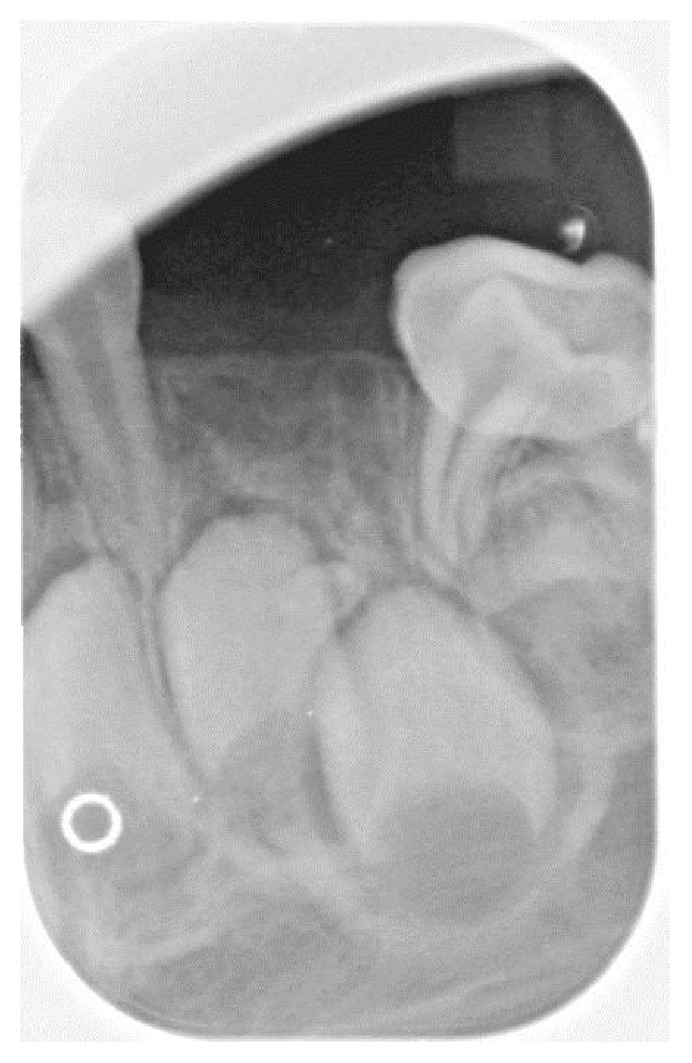
The tribal leader extirpated the lower left primary canine, but there appears to be a remaining tooth-like remnant.

**Figure 5:**
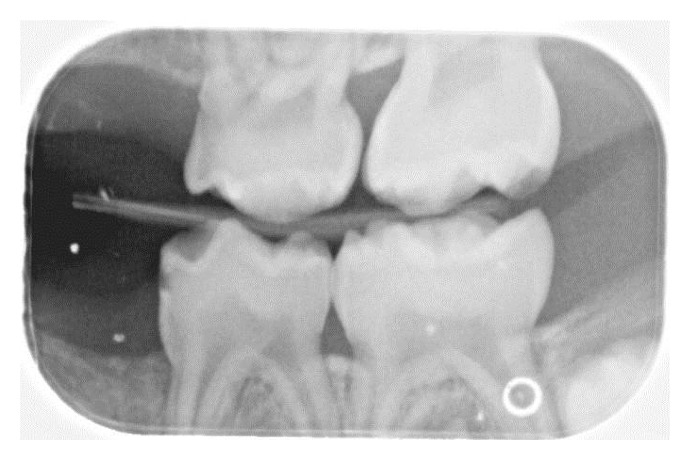
The radiograph demonstrates the absence of the maxillary and mandibular left primary canines.

**Figure 6:**
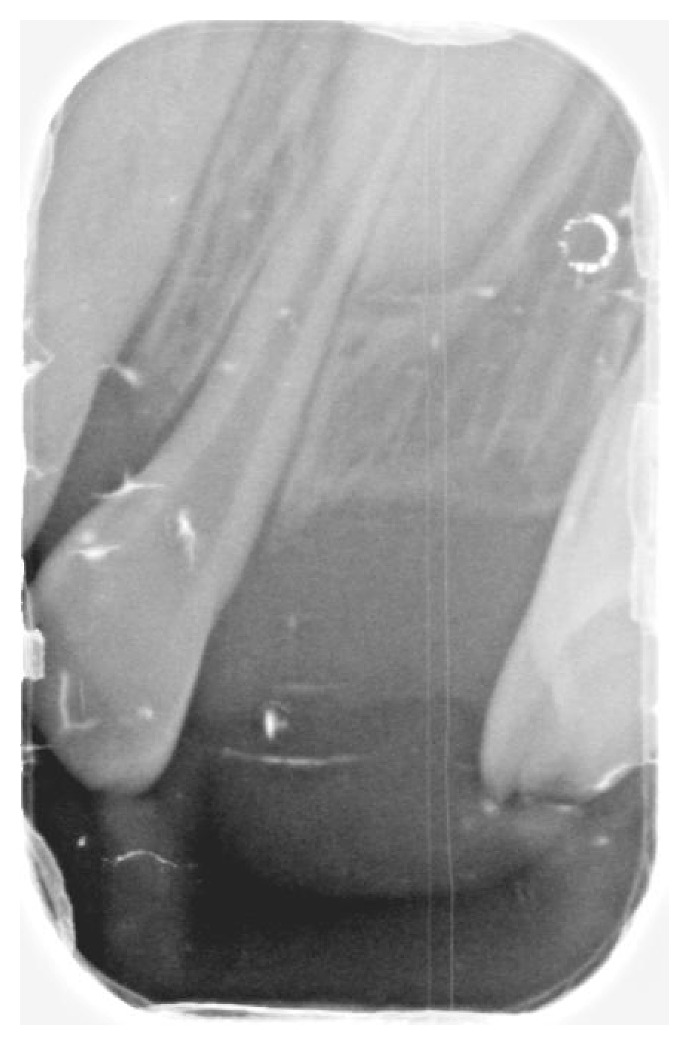
The periapical film confirms the absence of the primary maxillary left canine.

Radiographs confirmed what clinically appeared to be the complete absence of the maxillary left and mandibular right primary canines (63 and 83). Additionally, the right bitewing radiograph showed a suspicious area mesial to the maxillary right first primary molar (Figure 2). A follow-up periapical radiograph of this region was obtained, which demonstrated an unerupted dysmorphic maxillary right primary canine tooth (53) positioned ectopically near the mesial aspect of the maxillary right first primary molar (Figure 3). Upper right and lower left periapical films have atypical presentation distal to the upper right lateral permanent incisors and between permanent lower left canines and lateral incisors. It appears that the tribal leader failed to completely extirpate the developing upper right maxillary primary canine (53), and probably the lower left primary canine (73) due to the continued development of the upper right maxillary primary canine (53) and what appears to be a developing tooth-like remnant on the lower left primary canine area (73) (Figure 4). Additional radiographs confirmed the absence of the maxillary and mandibular left primary canines (Figures 5 and 6). These areas will be followed for the possible formation of supernumerary teeth or odontomas.

Caries were also noted on the mandibular left first primary molar and the mandibular right first primary molar (Figure 2). All findings and a treatment plan to restore the carious lesions were presented to the caregiver. Regarding the missing primary canines, an orthodontic plan was developed to follow the patient's growth and development and refer the patient to an orthodontist for evaluation at age seven. The permanent canines, lateral incisors, and first premolars will be monitored for possible hypoplasia secondary to trauma caused by the extirpation of the primary canines.

## 3. Discussion

In parts of eastern Africa, specifically Ethiopia, Uganda, Tanzania, Somalia, and Sudan, infant oral mutilation (IOM) is commonly practiced among tribal healers [7]. This practice is known among traditional healers as *ebinyo*. *Ebinyo*, also called *ebino*, *lugbara*, and *nylon*, refers to a practice in which the primary canine tooth follicles of infants are extirpated, without anesthetic, by traditional healers or elders using unsterilized instruments [8]. These instruments include bicycle spokes, knives, razor blades, hot needles, and even fingernails. As one can imagine, the use of these instruments has the potential to result in medical complications and infections. Unlike tongue and lip piercings, the most common forms of oral mutilation in the United States, oral mutilations performed in these rural areas are for perceived medical benefit. It is believed that the canine tooth follicles are associated with headache, nausea, and vomiting—all common symptoms reported among African children. Upon removal, the canine tooth follicles have a worm-like appearance—further strengthening the belief that the canine tooth buds are symptom inducing and that *ebinyo* is of therapeutic value [9].

Research reveals that IOM is performed on infants with a median age of five months. Coincidentally, children are beginning to teeth and are often being weaned from breastfeeding at this age. The transition from breast milk to probably unclean water and food sources often leads to severe dehydration among African children. Common symptoms of dehydration include headache, nausea, and vomiting, while teething may also result in irritability and swollen gums. Moreover, primary canine follicles often appear larger in the mouths of dehydrated infants. Joseph Hurlock was a European surgeon who promoted incising the gingiva over erupting teeth to relieve pain [7]. This practice lost popularity in the 20th century, but it is possible that traditional healers adopted this practice from colonial dentists working in Africa.

In other studies involving IOM, sequelae that are consistent with the findings of this case and additional consequences of primary tooth bud extirpation are noted. In one analysis by Holan and Mamber [8], 59 children who were suspected to have had their primary canine tooth buds extirpated were examined clinically to assess the long-term consequences. Their findings indicated that common complications include missing mandibular primary lateral incisors, hypoplasia of adjacent primary and permanent teeth, dilacerations of primary canines, failure of development of the permanent canine, and early eruption of the permanent dentition. Another case involving three siblings from Uganda described by S. N. Dewhurst and C. Mason reports supernumerary teeth apical to the mandibular right permanent canine in one of the siblings [8]. In a study comparing Israeli children and those of Ethiopian immigrants living in the same community, an association was noted between the absence of canines and the prevalence of dental defects. The absence of canines and presence of dental defects occurred in 60% of the Ethiopian population compared to dental defects in only 7–12% of the Israeli population. Only 10% of the Israeli children were missing canines [3]. Although it is challenging to study the long-term consequences associated with IOM due to limited cohorts of affected individuals, it should be noted that there are acute risks associated with the practice.

In a study conducted by Accorsi et al. [10], it was determined that one-fourth of the children in Northern Uganda who were hospitalized in 1999 died as a result of *ebinyo*. The most common complications leading to hospitalization are septicemia and severe anemia. In the same study, *ebinyo* ranked third behind meningitis and malnutrition in disease-specific case fatality rate. There have been several attempts to educate parents about the dangers associated with IOM, but the results are disappointing. In 1982, following a health program in Northern Uganda to prevent *ebinyo*, mothers began taking their children to the hospital immediately after IOM instead of avoiding the practice altogether. Even groups that immigrate, such as the Ethiopian community in Israel, continue the practice of *ebinyo* twenty years later, demonstrating a desire to preserve culture and reject western medicine [3]. Despite educational programs, *ebinyo* is still practiced in parts of Africa, and since most children survive the procedure, the false notion that there is medical benefit associated with IOM is, unfortunately, perpetuated.
